# Non-linearities in Theory-of-Mind Development

**DOI:** 10.3389/fpsyg.2016.01970

**Published:** 2017-01-04

**Authors:** Els M. A. Blijd-Hoogewys, Paul L. C. van Geert

**Affiliations:** ^1^INTER-PSYGroningen, Netherlands; ^2^Department of Clinical and Developmental Psychology, Faculty of Behavioural and Social Sciences, University of GroningenGroningen, Netherlands

**Keywords:** Theory-of-Mind, ToM_Storybooks, development, dynamic_systems_theory, non-linearities, anomaly

## Abstract

Research on Theory-of-Mind (ToM) has mainly focused on ages of core ToM development. This article follows a quantitative approach focusing on the level of ToM understanding on a measurement scale, the ToM Storybooks, in 324 typically developing children between 3 and 11 years of age. It deals with the eventual occurrence of developmental non-linearities in ToM functioning, using smoothing techniques, dynamic growth model building and additional indicators, namely moving skewness, moving growth rate changes and moving variability. The ToM sum-scores showed an overall developmental trend that leveled off toward the age of 10 years. Within this overall trend two non-linearities in the group-based change pattern were found: a plateau at the age of around 56 months and a dip at the age of 72–78 months. These temporary regressions in ToM sum-score were accompanied by a decrease in growth rate and variability, and a change in skewness of the ToM data, all suggesting a developmental shift in ToM understanding. The temporary decreases also occurred in the different ToM sub-scores and most clearly so in the core ToM component of beliefs. It was also found that girls had an earlier growth spurt than boys and that the underlying developmental path was more salient in girls than in boys. The consequences of these findings are discussed from various theoretical points of view, with an emphasis on a dynamic systems interpretation of the underlying developmental paths.

## Introduction

### Theory-of-Mind

The child’s Theory-of-Mind (ToM) is an important condition for showing socially adequate behavior (Astington and Jenkins, 1995; Imuta et al., 2016). ToM refers to the ability to attribute mental states **–** such as beliefs, desires, intentions, emotions, and perceptions **–** to oneself and others and to use these mental states in understanding, predicting, and explaining the behavior of oneself and others (Premack and Woodruff, 1978**;** Mitchell, 1997).

For instance, a child comprehends that if Sam thinks his soccer ball is in the garage (a belief), he will look in the garage for this soccer ball (the consecutive action), even though the soccer ball may in reality be in the garden. A typical five-year-old who is questioned about the actions of Sam and who also knows the true location of the soccer ball will be able to predict the action of Sam correctly. A typical three-year-old, however, will not be able to do so: he will most likely say that Sam will look in the garden. The 3 year old cannot distance himself from the knowledge of the true location, and he does not comprehend that others can hold beliefs that do not match reality as he sees it. He does not grasp false beliefs yet.

Numerous studies have suggested that a distinct change occurs in understanding these false beliefs between the age of 3 and 5 years old (for meta-analyses on false beliefs see Wellman et al., 2001; Liu et al., 2008). Though considered a cornerstone ability, ToM is far more than only false belief understanding and also extends beyond the 3–5 age period. Infants already possess ‘implicit’ mindreading capacities (Slaughter, 2015), treating themselves and others as intentional agents and experiencers; while older children understand lies and deception (Peterson and Siegal, 2002). Research shows that ToM development even prolongs into late adolescence (Dumontheil et al., 2010; Vetter et al., 2013; Valle et al., 2015).

### Developmental Sequences in Theory-of-Mind

Research has shown that ToM develops in normally developing children according to a particular, age-related sequence. It evolves from a simple desire theory to a complete belief-desire theory, from true beliefs to false beliefs, and from the understanding of first-order beliefs to second-order beliefs (Wellman, 1990). Deviations from this normal developmental path have been used in describing ToM difficulties of, for instance, children with autism (Baron-Cohen, 2000; Peterson et al., 2012). Relatively little research has been done on the effect of gender on ToM development, but, some studies have found an advantage for girls (Charman et al., 2002; Calero et al., 2013), including the finding that the association between ToM and prosocial behavior is stronger in girls than in boys (Imuta et al., 2016).

Wellman and Liu (2004) looked into the conceptual changes of different ToM aspects, using the ToM Scale. They found a consistent progression of conceptual achievements that pace ToM understanding in normally developing children: diverse desires > diverse beliefs > knowledge access > false belief > hidden emotion (Wellman, 2012, 2014). Wellman and Liu (2004, pp. 536) argue that the ToM developmental order is not one of addition or substitution, but one of modification or mediation. Initial insights broaden or generalize into later insights, following orderly conceptual progressions. A conceptual development has recently also been demonstrated for more advanced ToM tasks (Osterhaus et al., 2016).

### Temporary Regressions in Development

One can question how these ToM generalizations come about. Is there a gradual development or are there temporary accelerations, delays or even regressions observable during ToM development? Temporary regressions imply that children can have a temporary relapse before a newly acquired ability consolidates. This phenomenon is often referred to as U-shaped or N-shaped development (Siegler, 2004; Zelazo, 2004).

Temporary regressions have been found in a variety of domains, including motor and verbal development (Gershkoff-Stowe and Thelen, 2004; Swingley, 2009), non-verbal symbol learning (Namy et al., 2004), face perception (Cashon and Cohen, 2004), false belief understanding (Bernstein et al., 2011), intent-based moral judgments (Margoni and Surian, 2016), creativity, reasoning, and auditory localization (for an early collection of studies, see Strauss and Stavy, 1982; for modeling this U-shaped development, see Morse et al., 2011; for a recent overview, see Pauls et al., 2013).

In addition to temporary regressions, developmental curves may also show accelerations, which are often the hallmark of rapid changes that mark developmental transitions (see for instance Fischer and Bidell, 2006). Such developmental transitions are likely to be preceded by temporary regressions (Van Geert, 1991; Fischer and Bidell, 2006).

Temporary regressions, accelerations, and temporary plateaus are examples of non-linear forms of developmental change. One can question to what extent such non-linearities also apply to the development of ToM.

### Measuring Potential Non-linearities in Theory-of-Mind Development

In order to be able to observe potential regressions and accelerations in ToM development, one should take two issues into account. First, since ToM development does not solely depend on the development of false belief understanding, the research instrument used to measure ToM development should involve a variety of ToM components, like emotion understanding, belief understanding linked to actions (such as false beliefs) and emotions, desire understanding linked to actions and emotions, and relevant ToM precursors and associated abilities, like the understanding of the difference between mental and physical entities (Wellman, 1990). For that purpose, we developed the ToM Storybooks (Blijd-Hoogewys et al., 2008; see also “Materials and Methods”). Second, as there is no convincing evidence that by the age of six ToM is fully acquired (e.g., Hala and Carpendale, 1997; O’Hare et al., 2009) and stable, research should aim at a considerably broader age range, for instance up to 12 years old and even older (until adulthood).

At first glance, a time-serial design would be superior in order to follow the changing level of ToM over the course of developmental time. This is a design with as many measurements as are needed to capture the temporary and often non-linear forms of change characteristic of a particular developmental phenomenon in individual children (Steenbeek and van Geert, 2002; van Geert and Steenbeek, 2005). However, such a method also brings along considerable logistic problems. Children need to be tested repeatedly over an extended period. Also, since so few research has focused on the dynamics in ToM development, it is hard to predict at what time intervals children should be tested in order to find evidence of developmental phenomena such as accelerations and decelerations, transitions and temporary regressions.

Meanwhile, a cross-sectional design might provide a preliminary answer to the question of age-related changes and potential critical points in ToM in the population, and is a first step toward future time-serial research of developmental paths, as they occur in individual children. However, it is becoming a well-established fact that a developmental curve based on cross-sectional data should never be automatically identified as a representation of individual developmental curves (e.g., as the curve that applies to the ‘average’ child or the majority of children), until it has been empirically demonstrated, with the aid of a sufficient number of individual developmental curves, that individual-based curves are statistically and structurally similar to the developmental curve based on group data. This latter condition is known as the homology or ergodicity condition, and is rather unlikely to occur in the case of developmental processes (Molenaar and Campbell, 2009).

### Using Cross-Sectional Data to Tap Potential Non-linearities in Theory-of-Mind Development

Cross-sectional growth curves may serve yet another purpose than serving as first approximations of phenomena that require further scrutiny by means of individual time-serial designs. In this article, we propose an alternative perspective on the interpretation of cross-sectional data, which is based on the obvious fact that making a test amounts to the performance of a particular task, in which the child is asked to solve a particular series of problems, framed in a particular format.

In general, there exist various ways in which task performance can be used to obtain information about children’s development and about what they have learned from experiences. One way is to ask children to perform a familiarized and trained type of task independently and without help, such as solving math problems that are framed in a familiar and trained format, to see how much they have learned from their math lessons. Another way is to ask children to perform a particular task that lies beyond the child’s capability to solve this particular task independently, and to provide the child with help for doing so. This is the approach taken in dynamic testing (Grigorenko and Sternberg, 1998). A third possibility is to confront the child with a novel task, and to observe how far the child can get if it has to rely entirely on its own capabilities, eventually pushing the child to its limits by giving counter-suggestions or by repeatedly asking the same sort of question. The novel task can be novel in terms of content, and/or in terms of the problem format. In this case, the level of capability, development or learning is defined as the ability to transfer knowledge or skills from one context (e.g., the context of spontaneous daily experience and actual behavior) to another context, which can be of various kinds.

We contend that the administration of a ToM test that the child is unfamiliar with, amounts to observing a child’s developmental capabilities by providing it with a novel task content and format. A cross-sectional administration of the test that is likely to be a novel context for virtually every tested child can thus be seen as a way of mapping individual and age-related variability in the way children process this novel task, by means of a highly simplified measure, which is the set of sub-scores and the total test score of each individual. In this way, a cross-sectional procedure provides yet another perspective on a complex phenomenon, namely children’s development of ToM that can only be fully understood if it is viewed from a wide variety of perspectives. Hence, a cross-sectional design provides an answer to the question of age-related changes in how children transfer their knowledge about ToM that functions in daily contexts of spontaneous activity to a new context, namely that of explicit verbal questions and pictorial representations. It should be noted that a repeated administration of the same test may provide yet another kind of information, namely differences between children in their ability to spontaneously learn from repeatedly performing the same task (without feedback; Blijd-Hoogewys et al., 2010).

### Statistical Indicators of Non-linear Developmental Phenomena

In order to describe changes in development, different fitting models can be used to represent the general underlying trend. In research, linear or quadratic models are often used. Unfortunately, such models do not sufficiently take local deviations of the distribution of data into account. This may lead to over- and underestimations of the expected average scores in certain age periods.

In contrast, non-parametric models, like Loess (or Lowess) estimate smoothing procedure, follow local distributions of data as reliably as possible. They apply a locally weighted least squares estimate, and are commonly used as smoothing techniques (see for instance Simonoff, 1996). Such non-linear techniques can be of substantial value for testing non-linear changes even when applied to cross-sectional data. Examples of such non-linear changes are accelerations, decelerations, and temporary regressions. Additional indicators of developmental transition are changes in the skewness of the distribution, temporary changes in growth rate and changes in variability (van Geert and van Dijk, 2002; Bassano and van Geert, 2007; Van Dijk and van Geert, 2007).

Changes in the skewness of the distribution over time may provide information about alternations between periods of relative stability (zero skewness) and periods of rapid change beginning with a minority of rapid developers (positive skewness) heading toward a new period of relative stability with a minority of children lagging behind (negative skewness).

A temporary change in growth rate can be demonstrated in the form of marked oscillations in the first derivative of the developmental curve, which represents the rate of growth at that point. A particularly strong instance of change in the growth rate occurs in the form of a temporary regression (a local dip), where the growth rate temporarily drops down to negative values. Since the changes in skewness over time are related to accelerations in the growth of the developmental phenomenon at issue, we expect to find a certain level of coherence between the first derivative of the non-linear ToM growth curve and the change of skewness over time.

Change in variability, the third indicator of developmental transition discussed in this article, can be observed as intra- and inter-individual variability. A temporary increase in the intra-individual variability is considered a strong indicator of a developmental transition (van Geert and van Dijk, 2002). However, such an indicator can only be used in repeated measures designs. Inter-individual variability, which is applicable to cross-sectional data and which is expressed in terms of standard deviation over a certain period of time, might also temporally increase during a transition.

Although these three indicators are likely to be correlated, if they are indeed indicative of an underlying developmental transition, they are, in principle, independent of one another. For instance, an acceleration in the group-based growth curve might occur without any change in intra-individual variability, or without any change in skewness.

### Aims, Hypotheses, and Research Questions

In the sections above, we have provided evidence for the occurrence of developmental regressions and various other non-linearities in the development of a wide variety of skills and forms of knowledge. So far, no studies have explicitly looked at such eventual non-linearities in ToM development. The objective of this study is to investigate whether there occur developmental regressions and other non-linearities during ToM development in childhood.

In addition, we have seen that ToM is not a monolithic ability. It consists of various sub-abilities, each with their characteristic developmental timing. Hence, if ToM development is characterized by non-linearity, it is likely that the forms of these eventual non-linear properties will differ between various aspects of ToM.

Finally, gender differences have been found in ToM development, with girls having a slight advantage over boys. The question is whether this difference is also observable in the form of the cross-sectional developmental trajectories in boys and girls, i.e., whether eventual non-linearities in the curves have a gender specific timing or form.

Given the present state of our knowledge, all these issues amount to open questions. So far, there is no theory from which the answers to these questions can be predicted and that allows us to formulate these questions in the form of hypotheses. In this article, we will formulate a dynamic model of ToM development that might serve as a first attempt toward such a theory.

To summarize, our research questions are as follows: (1) Are there non-linearities in the cross-sectional growth curve of ToM in the form of temporary regressions and accelerations? (2) If such non-linearities are observed, are they real or ordained due to statistical or sampling artifacts? (3) Are eventually observed non-linearities supported by additional indicators of non-linear change as described above? (4) Are there differences in the eventually observed non-linearities between (4a) the various aspects of ToM as represented by the ToM sub-scores, and (4b) boys and girls?

In order to answer these questions, we follow a cross-sectional design, for reasons explained in the section on tapping eventual non-linearities in ToM development. We use the ToM Storybooks, an instrument that incorporates a variety of ToM components. In order to describe the possible temporary regressions and accelerations in ToM development, we use techniques that also look at additional indicators for developmental transitions: changes in the skewness of the distribution, temporary changes in growth rate and changes in variability.

## Materials and Methods

### Ethics Statement

The ethical committee of the University of Groningen approved this study and written consent was obtained in advance from parental guardians. The methods were carried out in accordance with the approved guidelines. Minors were involved. Their parents were asked for written consent.

### Participants and Setting

We tested 324 children. The ages ranged from three up to and including 11 years, with approximately the same number of boys and girls per age range (**Table 1** for the age distribution).

**Table 1:** Age distribution of sample being administered the Theory-of-Mind (ToM) Storybooks (*N* = 324).

	Age (in years)							
	3	4	5	6	7	8–9	10–11	Total
Boys	32	31	31	31	15	14	13	167
Girls	29	24	32	26	16	12	18	157
All	61	55	63	57	31	26	31	324

The children came from preschools, kindergartens, and elementary schools, from both provincial and urban regions in the Netherlands. All children had a Dutch linguistic background, and did not have language acquisition problems that could have hampered their performance on the tasks (for the role of language in ToM development and ToM performance: Milligan et al., 2007; de Villiers and de Villiers, 2014; Ebert, 2015). Two Dutch language tests were used, depending on the age of the child. For 3–6 year olds, the Reynell was administered (test for receptive language comprehension; Van Eldik et al., 1997); and for 6–9 year olds, the TvK (Taaltest voor Kinderen, Language Test for Children: subtests ‘vocabulary’ and ‘sentence construction’; Van Bon, 1982) was used. Language scores were available for 249 children (Reynell: *n* = 170, TvK: *n* = 79). Those children who did not receive a language test were older than 6 years and judged as having appropriate language skills by their teachers. Thirteen percent of the children came from a lower social background, distributed over the whole age range. This percentage corresponds with the percentage as known from the Dutch National Bureau of Statistics, at time of the research.

### Measure

Children’s ToM knowledge was tested with the ToM Storybooks, version Sam (Blijd-Hoogewys et al., 2008). It is a comprehensive test, composed of multiple tasks, that measures a variety of ToM components and associated aspects. The ToM tasks are incorporated in short stories. These stories are illustrated with full color pictures and enlivened by the use of cuddly patches of fur, toy doors that can be opened, and magnetized emotion faces that can be placed on the characters. The test takes 40–50 min, including a short break (5 min of free play), also for the youngest age group (no significant effect of fatigue was found; Blijd-Hoogewys et al., 2008). Children experience the assessment as a ‘being read to’ activity, rather than a ‘being tested’ activity.

In total, there are 34 tasks spread over six storybooks in total. A maximum sum-score of 110 points can be obtained, which can be divided into five sub-scores (for example tasks, see Appendix A in Blijd-Hoogewys et al., 2008): (1) emotion recognition (maximum = 14 points), (2) distinction between physical and mental entities (real-mental, real-imaginary, and close impostors; maximum = 44 points), (3) understanding that seeing leads to knowing (maximum = 3 points), (4) understanding of desires (maximum = 17 points), and (5) understanding of beliefs (maximum = 32 points). The latter encompass tasks on standard belief, changed belief, not own belief, explicit false belief, false belief, inferred belief, and inferred belief control. In addition to providing a single, quantitative measure of the level of ToM ability, the ToM Storybooks also allow investigators to compare various relevant ToM components.

Each task incorporates one to five questions, including both test questions and justification questions. There are in total 74 binary test questions and 18 justification questions. The answers to the test questions are coded as correct or incorrect (1 or 0 points; maximum of all test questions = 74). The justification questions result in 2, 1, or 0 points, depending on the amount and correctness of the mental state terms spontaneously used by a child (maximum of all justification questions = 36). In order to evaluate the justifications, a category system is used (for more details, see the Appendices in Blijd-Hoogewys et al., 2008).

Since the ToM Storybooks is a comprehensive test, no other ToM measures were included in this study. The test has good psychometric qualities. The internal consistency [Cronbach’s alphas: ToM total score = 0.95, ToM sub-score 1 (emotion recognition) = 0.83, ToM sub-score 2 (physical/mental) = 0.88, ToM sub-score 3 (seeing knowing) = 0.51, ToM sub-score 4 (desires) = 0.84, ToM sub-score 5 (beliefs) = 0.89], test-retest reliability (*r* = 0.86, *p* = 0.001 for typically developing children, *r* = 0.98 for children with PDD–NOS), inter-rater reliability (Cohen’s Kappa = 0.81–0.97), divergent and convergent validity are good (see also Blijd-Hoogewys et al., 2008, 2010). The ToM Storybooks has been translated in different languages, such as English, Finnish, French, Italian, and Spanish; and it has been standardized on two European populations, namely Dutch children (Blijd-Hoogewys et al., 2008) and Italian children (Molina and Bulgarelli, 2012; Bulgarelli et al., 2015).

### Procedure

All subjects were individually tested in a quiet room at school. Test administrators were carefully instructed to follow standard procedures. For practical reasons, kindergarten children were tested at home. If necessary, the parent was allowed to be present during testing but was requested not to interfere. The justification questions were judged later on. The inter-rater reliability of the justifications is known to be high (Cohen’s Kappa = 0.81–0.97, see Blijd-Hoogewys et al., 2008). The few differences left were unanimously agreed on after discussion by four researchers.

### Data Analysis

In order to acquire insight in non-linear changes in ToM development, we used a descriptive non-parametric method, namely Loess curve smoothing (Simonoff, 1996). Next to that, we used random permutation techniques, and more generally, Monte Carlo analyses, which are assumption-free techniques (Kroese et al., 2014). Wellman et al. (2001) have argued for the use of more assumption-free techniques, such as bootstrap methods, in ToM research. It entails a simulation of the test statistic at issue (e.g., a particular numerical indicator of change or of non-linearity) as based on the null hypothesis, which can be compared to our empirical ToM data (Good, 2001; Todman and Dugard, 2001; Manly, 2007).

## Results

### Non-linearities in ToM Sum-Scores

**FIGURE 1:**
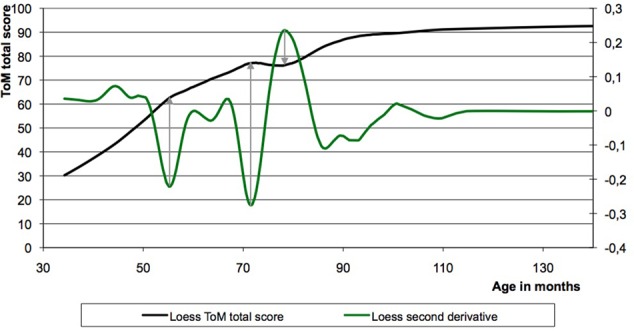
**The Loess fitting curve of the Theory-of-Mind (ToM) sum-score data plotted versus age displays a non-linearity.** Based upon the second derivative of this curve three points of developmental interests were found, namely at 56, 72, and 78 months.

The Loess smoothed curves of the ToM sum-scores (maximum = 110 points) reveal three points of developmental interest (**Figure 1**; see also Blijd-Hoogewys et al., 2010). To determine the exact timing of these points, minima and maxima of the second derivative (acceleration of growth) of the developmental curve were inspected. The most marked inflection points are seen at 56 months (4 years and 8 months), 72 months (6 years), and 78 months (6 years and 6 months). The second inflection point (72 months) is followed by a dip in the curve, which shows its deepest point at 78 months. This dip is a temporary regression or local U-shaped age curve. It is the most striking deviation from monotonicity in the non-linear developmental curve based on the data from boys and girls taken together. More detailed analyses can be found in the section ‘Non-linearities in ToM sub-scores and in gender based sub-groups.’

### Is the Temporary Regression at 72–78 Months Real?

It should be checked whether the non-linearity in the form of a temporary dip is not the result of inadequate selection procedures or of statistical artifact, such as accidental sampling effects or the influence of specific, biased or incompetent test administrators.

To begin with, inadequate selection procedures are highly unlikely since the selection procedure was carried out with the utmost care and selection criteria were uniform over all ages. Second, it is unlikely that the non-linearity in the form of a temporary regression (a dip) is a statistical artifact. In order to demonstrate this, the null hypothesis was tested that the generic curve underlying the data is actually a monotonically rising curve and that the dip is due to accidental sampling variations. The latter could amount to an accidental overrepresentation of low scoring individuals. In order to test this possibility, we calculated the best fitting monotonic growth curve and a regression model for the variances. Since we had no prior assumption about where a non-linearity, in the form of a temporary regression, in ToM ability should occur, we tested for the accidental occurrence of an apparent temporary regression anywhere along the time interval. Because a theoretical expectation about the length of the temporary regression is also lacking, the null hypothesis was also tested for time windows of different length. By means of a Monte Carlo technique, we calculated the probability that the null hypothesis model yields a temporary regression, comparable to the observed one. The pattern of probabilities supported the conclusion that it is unlikely that the observed temporary regression is an accidental sampling effect of an otherwise continuous, monotonically rising simple curve (Monte Carlo, *p* = 0.01 through *p* = 0.05, depending on the length of the tested interval). Another indicator for non-linearity in the form of a temporary regression, namely negative slope (over intervals of variable length), provided converging evidence (Monte Carlo, *p* = 0.02).

Next, we checked if particular test administrators caused the non-linearity in the form of a temporary regression (dip). We defined eight groups of data sets by leaving out the data of one particular test administrator at a time. If the temporary regression is due to an anomalous test administrator, it should disappear in the dataset from which this particular person is lacking. We repeated the statistical procedure described above for each of the reduced data sets. The resulting p-values showed that the dip remained significant for each of the reduced data sets (Monte Carlo, *p* < 0.001 through 0.05).

In summary, neither selection errors, nor accidental sampling errors nor a deficient test administrator can account for the occurrence of the observed non-linearities.

### Non-linearities in ToM Sub-Scores and in Gender Based Sub-Groups

**FIGURE 2:**
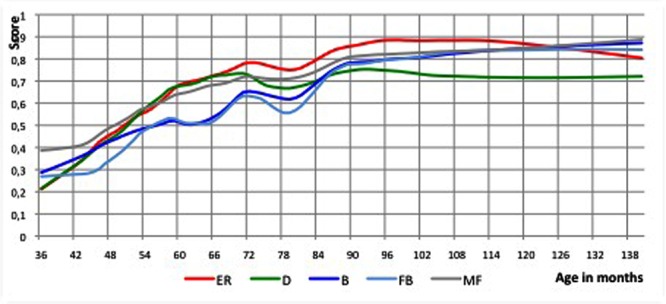
**Loess curves of the ToM sub-scores plotted versus age.** All ToM sub-scores showed dips at the same ages as the dips found for the ToM sum-score. ER, Emotion Recognition; D, Desire; B, Belief; FB, False Belief; MP, Mental Physical. The deepest point of the dip based upon the ToM sum-score is pointed out with the arrow and dotted line.

We checked whether the main temporary regression (a dip at 72–78 months) is observable in all five ToM sub-scores and preferably in the core ToM sub-scores (on desires and beliefs). For this purpose the ToM sub-scores were rescaled, to make comparisons easier (otherwise they would have different maximum scores). Loesses with a 20% window size were calculated. All ToM sub-scores showed dips at roughly the same age (Monte Carlo, *p* = 0.01; see **Figure 2**).

The curves of the ToM sub-scores showed the same characteristics as that of the ToM sum-score, with start and end of the major dip at roughly the same age as the dip based upon the ToM sum-score (start: respectively, 71–74 months vs. 72 months; end: respectively, 77–79 months vs. 78 months). The sub-score on false beliefs displayed the steepest dip.

**FIGURE 3:**
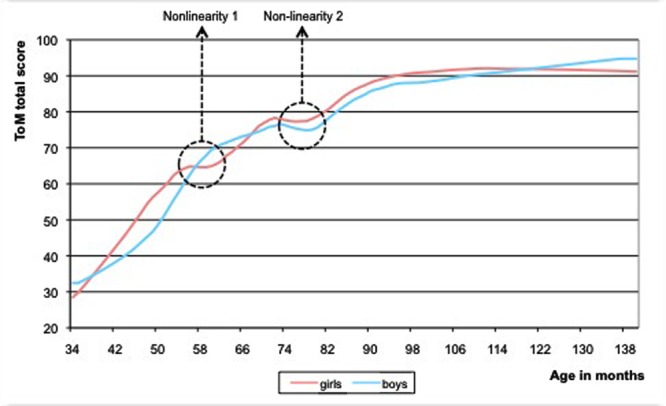
**Loess curves of the ToM sum-scores plotted versus age, for boys (black line) and girls (gray line).** The girls show two non-linearities, the boys only one.

Second, we looked whether there are gender differences. On average, girls had slightly higher ToM sum-scores than boys (*M* = 71.71 versus *M* = 68.73, respectively; independent samples *t*-test, *p* = 0.098). The variance hardly differed between both sexes (20.82 and 20.44) and is considered equal (Levene’s test, *p* = 0.749). When we divided the group in three age groups, however, (*n* = 87, <54 months; *n* = 119, 54 < 78 months; *n* = 118, ≥78 months), we found the gender difference to be significant for the youngest and oldest group (*p* = 0.05); and the variances within these two age groups were not equal (Levene’s test, *p* = 0.01 and *p* = 0.05, respectively). Subsequently, we compared the Loess curves for both genders (**Figure 3**). The girls showed two non-linearities: an increase between the fourth and fifth year, followed by a plateau (first temporary regression) and then again a growth spurt between the fifth and sixth year, followed by a dip (second temporary regression) and ending with an ultimate growth spurt. The boys showed only one non-linearity, namely a dip that was more pronounced than the simultaneous dip of the girls. Through slope hunting techniques, we investigated the statistical significance of these dips in the null hypothesis model. The dip of the boys was significant (Monte Carlo, *p* = 0.007). The dip of the girls (their second temporary regression) was more flat and did not reach significance (Monte Carlo, *p* = 0.20). However, this dip appeared around 3 months earlier than in boys. If we reckon with the fact that ToM develops earlier in young girls than in young boys (Charman et al., 2002), the earlier appearance of the dip in the girls seems a meaningful phenomenon. The probability that the occurrence of a dip of this magnitude, appearing up to 3 months earlier, but not later than in the boys is unlikely to be accidental (this difference is statistically significant; Monte Carlo, *p* = 0.03).

### Skewness and Variability as Additional Indicators of an Underlying Transition

In the introduction, we discussed three qualitative indicators of developmental transition, namely skewness, temporary changes in growth rate and change in variability. Before further analyzing the developmental ToM pattern, we first wished to determine whether the hypothesized properties stated are indeed characteristic of a developmental transition of the kind we now expect to find in the ToM data. In order to do so, we mathematically simulated a transition model in order to check whether the expected qualitative indicators occur. A good example of a developmental transition is a two-step growth process (for details on how such models can be specified and simulated, see Van Geert, 1991, 1994; a two-step growth process can easily be extended toward a three- and more-step model if needed). This transition model can be found in the Supplementary Material.

**FIGURE 4:**
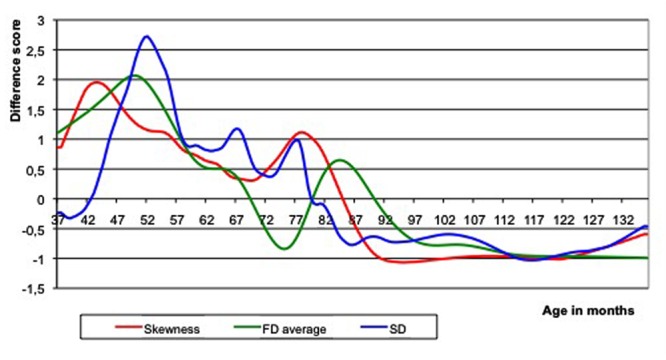
**Loess curves of the three qualitative indicators of developmental transition based on empirical data.** A mixture between a two-step and a three-step growth process is obvious. There are two peaks, both in the skewness and in the first derivative curve. FD, first derivative (or growth rate); SD, standard deviation (or variability).

**Figure 4** shows the Loess curves with a 30% window of the skewness, growth rate, and variability of the real ToM data. A mixture between a two-step and a three-step growth process is apparent. There are two large peaks, with a smaller peak in between, most clearly observable in the variability measure (standard deviation) and less in the other two measurements. The qualitative similarity with the model simulation of a two-step process is striking. There are two peaks, both in the skewness and in the first derivative (i.e., growth rate) curve. As is the case in the simulation, the peaks of skewness largely coincide with those in the first derivative (growth rate), and the skewness peaks come somewhat earlier than those of the first derivative. The covariance of the series is 0.88, which is comparable to (and even higher than) the high covariance that the simulation model predicted (0.70).

Before concluding that the skewness and first derivative data support the notion of a two-step developmental process, we need to know what the probability is that a similar co-variation of skewness and first derivative curves can be obtained if the underlying statistical variation of the sum-scores is in fact symmetrical across age (and not varying systematically, as hypothesized). This null hypothesis model can be tested by generating random series of sum-scores based on a normal distribution model, with means equal to the successive values of the non-linear growth curve and standard deviations equal to the observed standard deviation of the residuals. Only 2 out of the 200 simulated series had a covariance greater than or equal to the observed covariance (*p*-value is ∼2/200, i.e., *p* = 0.01). We can thus conclude that the skewness data provide further independent evidence for the existence of at least a two-step process in the development of ToM.

### Is There a Two or Three-Step Developmental Model?

As we mentioned before, girls evidenced a three-step development and boys more a two-step development (**Figure 3**). However, it is highly probable that also boys show a three-step development. It can be hypothesized that the first transition is observable only in girls, because of differences in major parameters – in particular the value of the main parameter, which is the growth rate – and not because of differences in the underlying variables affecting the growth of ToM.

**FIGURE 5:**
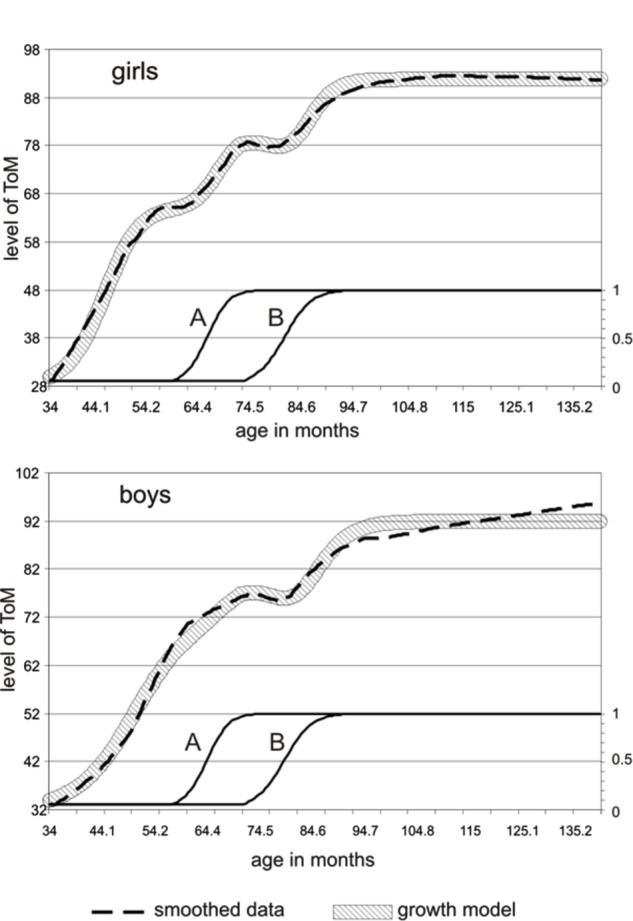
**A three-step growth pattern (broad striped line) fitted over the smoothed ToM sum-score data (interrupted line), taking into account the emergence of two underlying, supportive variables A and B.** The top graph shows the fit of girls, the bottom part shows the fit of boys. The underlying variables A and B are of the same magnitude and occur at the same age in both genders.

In order to show that this interpretation is indeed feasible, we fitted a three-step growth pattern of ToM knowledge, based on the emergence of two underlying, supportive variables, one around the age of 56 months (A) and another around the age of 72 months (B; **Figure 5**). These supportive variables are hypothetical and may for instance include executive functions, which are known to be an important facilitator in ToM functioning (for the relation between ToM and executive function see, e.g., Carlson et al., 2002, 2013; Devine and Hughes, 2014), also found across cultures (Wang et al., 2016).

The growth model that was fitted to the smoothed data is of the type described by Van Geert (1991, 1994), and by Fischer and Bidell (2006). It contains positive parameters, i.e., a supportive relationship, for the A and B levels and negative parameters, i.e., a competitive relationship, for the first derivative of the hypothetical A and B levels (which corresponds with the actual change in these levels). **Figure 5** shows the fit with the smoothed curves of boys and girls separately, based on underlying hypothetical variables A and B, which are of the same magnitude and occur at the same age in both sexes. **Table 2** shows the values of the model parameters.

**Table 2:** Values of the model parameters used for the dynamic systems growth model.

	Girls	Boys
Growth rate	0.15	0.10
Support from A	0.33	0.10
Support from B	0.24	0.16
Competition from the growth of A	0.00	0.00
Competition from the growth of B	-3.20	-2.50

A striking difference between boys and girls is that the parameter values cause faster growth and more effect of supportive and competitive variables in girls than in boys. The first discontinuity, a plateau, which is observable in the girls thanks to their higher growth rate, is in fact concealed in boys, as a consequence of their lower growth rate and lesser effect from the A-variable (which is a hypothetical variable emerging around the age of 4.6 years). The second discontinuity is observable in both girls and boys. Although the competitive effect of B on ToM is greater in girls than in boys, the observable effect is more salient in boys. This finding may lead to the conclusion that girls evidenced a three-step development and boys only a two-step development. However, in dynamic growth models, parameters often show non-linear co-variations, for instance competitive effects among variables can be masked by higher growth rates. The dynamic growth model (**Figure 5**) showed that the expression of the steps in the form of *observable* plateaus and marked dips may depend on the values of the growth parameters, in particular the value of the main parameter, which is the growth rate. It can be concluded that a dynamic growth model involving the effect of two variables affecting the growth of ToM, one occurring around the age of 56 months and the other around the age of 72 months, can account for the variety of non-linear phenomena observed in the data, including the differences and similarities, the plateaus and dips between boys and girls.

## Discussion

### Non-linearities in the Development of ToM: One or Two Temporary Regressions

Our findings support the general developmental view of ToM. Based on cross-sectional analyses of a ToM task that is new to the children, our results show that ToM increases with age – with the greatest increase between 42 and 56 months, that is between 3.5 and 4.7 years of age – and that it continues to develop after the age of six. The development before the age of four and a half is evidently monotonous. However, after this age non-linearities occur. Two temporary regressions – one around the age of 4 years and 8 months and one at the age of six to six and a half – are found not only in the ToM sum-score Loess curve but also in the ToM sub-score Loess curves.

The temporary regressions can be viewed as indicators of non-linearity in ToM development. We have demonstrated that the probability that the main temporary regression (a dip at 72–78 months) is either a statistical selection artifact or an experimenter artifact is very small. The application of additional indicators – skewness, growth rate, and variability – provided further support for the occurrence of a transition – or two transitions – in the development of ToM, as evidenced by an instrument that requires children to transfer their daily knowledge to a context of explicit verbal questions and pictorial representations. Also, the non-linearity found cannot be accredited to gender differences. Both boys and girls showed a marked regression around the age of six. However, girls also showed evidence for an additional earlier regression (a plateau), around the age of five.

There are different views on the manner in which ToM develops in preschoolers. For instance, one view implies continuous increases in ToM related processing abilities rather than radical conceptual shifts in understanding mental states (e.g., German and Leslie, 2000; Carlson and Moses, 2001; Birch and Bloom, 2004). A second view assigns central importance to the occurrence of a conceptual change. This change takes place between the age of three and four/five for simple ToM skills (Perner, 1991; Gopnik, 1993; Wellman et al., 2001; Wellman, 2014), and this conceptual development continues into more advanced ToM skills at the age of eight/ten (Osterhaus et al., 2016). Our data show a pattern of overall continuous increase, with a steep growth of ToM knowledge around the age of four, followed by a more continuous increase of ToM knowledge leveling off toward the age of five and interrupted by a temporary regression around the age of six, which occurs in boys and girls alike. Overall, we found boys and girls to follow the same developmental path. However, we also found some gender differences in ToM development. Such differences have seldom been reported in ToM research (for exceptions: Charman et al., 2002; Walker, 2005; Calero et al., 2013). In fact, most studies find no statistically significant differences between boys and girls, which might be due to the use of tests that are insufficiently capable of capturing subtle individual ToM differences (Baron-Cohen et al., 1997), or have insufficient statistical power. Our study included a more extensive sample than the majority of studies did. In addition, we employed statistical techniques that are sensitive to more subtle developmental patterns. Under such methodological conditions, eventual gender differences are more easily recorded from the data, not only in the appearance of ToM skills but also in the rate of ToM development. The early ToM growth in girls was more rapid than that of boys. Gender difference in the rate of ToM development has been hypothesized before by Baron-Cohen et al. (1997) and by Charman et al. (2002) who found that young girls have a ToM advantage, which disappears as children get older. Such a higher early rate of growth results in a greater likelihood of a later temporary standstill (Van Geert, 1994), which has indeed been demonstrated in our data, for girls showed two non-linear changes in the form of temporary regressions (a plateau and a dip), and boys only one (a dip). This is in correspondence with the scarce research on the effect of gender on ToM showing slight ToM advantages in both young girls (2.3–4.3 year olds, Charman et al., 2002) and more profound ToM advantages in older girls (6–8 year olds, Calero et al., 2013). The more rapid ToM growth in girls might be due to the fact that, from the beginning, girls are more focused on sociability. For instance, already in 1 day old neonates, a definite sexual dimorphism is observable (Connellan et al., 2000). Next to that, girls also have better verbal abilities than boys (Halpern, 2000), stronger syntactic abilities and a larger amount of social experiences (Charman et al., 2002). Language is considered an important factor in ToM functioning (e.g., de Villiers and de Villiers, 2014). Finally, there is some evidence that females show more pronounced responses of the mirror neuron system than males (Cheng et al., 2006); the mirror neuron system has been hypothesized to directly relate to ToM abilities in both children and adults (for a review see Oberman and Ramachandran, 2007).

### Potential Explanations for the Observed Temporary Regressions

In this article we reported the discovery of one or two temporary regressions, indicative of either a two- or three-step development. The literature on U-shaped growth and non-linear growth curves in general provides some hints on possible explanations.

The first explanation is that the non-linearities reflect a temporary conflict between competence and performance (Marcus, 2004). According to this view, the development of ToM competence follows in reality a monotonically rising function, but for some accidental reason, performance on ToM tests gets a little worse around the age of six, maybe because a particular performance component interferes negatively. The question is of course what this performance factor is. In addition, one may question whether this competence-performance distinction is relevant on the level of testable psychological functions. Dynamic systems theory, as advocated by the late Esther Thelen and her collaborators, makes no distinction between these two levels, and sees a temporary regression as a direct consequence of dynamic interactions between components that are responsible for the production of answers to ToM questions in specific problem contexts (Gershkoff-Stowe and Thelen, 2004). According to this view, there is no ToM in the sense of an identifiable, internal conceptual structure. All behavior is soft assembled, and temporary regressions reflect the “continuous changes in the collective dynamics of multiple, contingent processes” (Gershkoff-Stowe and Thelen, 2004, page 11).

Another point that we wish to re-emphasize is that, from a dynamical point of view, cross-sectional data based on test scores provide an answer to the question of how children transfer their daily probably non-discursive experiences to a context of repeated, explicit verbal questions and pictorial representations. From a dynamic systems point of view, all forms of knowledge expression reflect the process by which this expression has come about. In that sense, all information about development reflects the contextual conditions under which it has been obtained. It is thus possible that the non-linearities found in our study are a typical property of the current test conditions. However, this eventual context dependency does not reduce the developmental significance of the information obtained. The question is of course which aspect/aspects of ToM related knowledge and behavior is/are responsible for the observed non-linearities, in particular the temporary regression.

According to Brainerd (2004), temporary regressions in performance occur if a particular performance class – for instance the class of ToM related questions – is served by opposing strategies, or dual processes. It is conceivable that up to the age of six, the child has employed an intuitive and direct solution to ToM problems, while at around the age of six a new approach begins to emerge, which is more cognitive and reflective in nature (see also the hypothesis of embodied/enacted and explicit/reflective perspectives on other persons, e.g., Bohl and van den Bos, 2012; Fuchs, 2013; Gallagher and Varga, 2014). The emergence of a second strategy – for instance implying an explicit third person perspective as Fuchs (2013) has called it – requires a form of reorganization of components responsible for ToM performance, and the observed non-linearities are likely to reflect this reorganization (Feldman and Benjamin, 2004; Friend, 2004; Marcovitch and Lewkowicz, 2004; Rogers et al., 2004; Wewrker et al., 2004). That such non-linearities indeed occur as a consequence of continuous, long-term growth in a developing system has been demonstrated by modeling development, either by means of connectionist networks (Rogers et al., 2004) or by means of dynamic systems models of the type advocated by Van Geert, Fischer, and others (see Demetriou and Raftopoulos, 2004, for a discussion regarding U-shaped growth). In these models, long-term development is context-specific and dependent on dynamic interactions among many components – biological, cognitive, emotional, behavioral – that constitute the developing system (Van Geert, 1991, 1994, 1998; Fischer and Rose, 1994; Fischer and Bidell, 2006; Fischer and Van Geert, 2014). Relationships between the multiple components in a system can be supportive, competitive, conditional, or neutral. The dynamics of these relationships over time explain the emergence of phenomena such as accelerations, decelerations, and regressions.

Based on dynamic modeling and indirect evidence from brain development, neo-Piagetian theory predicts relatively major shifts in development around the age of 6 years, dependent on the context or content of the developmental function (Case, 1991; Fischer and Bidell, 2006). The shift is broadly associated with a marked increase in more reflexive, coordinated ways of thinking in contrast with the more intuitive, uni-dimensional ways of thinking that precede it. Although the application is purely speculative, it might be so that around the age of six the intuitive ToM judgment, which is considered to be largely based on biologically founded forms of empathy (Preston and de Waal, 2002) is supplemented by a more reflective, cognitive form of ToM reasoning (already constructed form age 4 onward; Low, 2015). In this regard, it has been shown that six-year-olds have little trouble assigning false beliefs to others, but only arrive at a truly interpretive ToM at the age of seven (Carpendale and Chandler, 1996; Lalonde and Chandler, 2002), however, ToM continues to develop and change throughout life (Moran, 2013; Vetter et al., 2013). Children with autism seem to have an implicit ToM deficit (Schuwerk et al., 2015). As predicted by the theories discussed earlier, this emergence of a new ToM specific strategy in typical development might explain the temporary regression found in our data. The fact that this regression was found for all ToM sub-scores supports this way of thinking.

The previous explanations all rely on the notion of distinctive, developmentally ordered strategies for solving ToM problems. In fact, there is supportive but indirect evidence of two ‘approaches’ to ToM: an intuitive (or automatic) and a reflective (or controlled) route (Lieberman, 2007). Indirect evidence for an intuitive, neuro-physiologically based understanding of ToM related properties of other persons comes from the rapidly growing literature on the neuronal systems that underlie the spontaneous understanding of human actions and psychological states of others. An example of such a system is the mirror neuron system (for a systematic review see Hamilton, 2013). It is hypothesized that through cognitively mediated routes people with autism are able to compensate for the lack of an intuitive ToM (Eisenmajer and Prior, 1991; Baron-Cohen et al., 1993; Dissanayake and Macintosh, 2003). It is a strategy they can only master if a verbal mental age of 11 years is attained (e.g., Happé, 1995). Typically developing subjects, on the other hand, use the direct biology-based routes as well as the more cognitive ones. Their understanding of ToM is a combination of approaches and strategies (Lieberman, 2007), the combination of which changes across development (Kobayashi et al., 2007). It is not unlikely that the temporary regressions found in our study reflect a major reorganization in the composition of strategies.

It should be noted though that the non-linearities found in our data need not reflect a difference in ToM understanding *per se*, but could reflect a developmental difference in other factors necessary for the task. For instance, attention, inhibition, and ‘curse of knowledge’ may play a role (e.g., German and Leslie, 2000; Carlson and Moses, 2001; Birch and Bloom, 2004). At the age of six, the development of executive functions undergoes its first active stage of maturation (Brocki and Bohlin, 2004). It is not unthinkable that this development also has consequences for the ToM development of children (Carlson et al., 2002). According to the emergence account, executive function is even considered a necessary condition for the acquisition of ToM understanding (Moses, 2001; Devine and Hughes, 2014). San Juan and Astington (2012) have even suggested that executive function and language abilities can aid the developmental step from an implicit to an explicit ToM. However, Osterhaus et al. (2016) found that advanced ToM abilities were not determined by information-processing capacities (such as executive control: working memory and inhibition), instead indicating conceptual development.

Finally, data collected on children with PDD–NOS, an autism spectrum disorder, (Blijd-Hoogewys et al., 2010) show a highly comparable dip in ToM scores. However, in accordance with the developmental delay in ToM typical of such children, the dip occurs at a slightly later age than in the typically developing children. This delay in the timing of the dip supports the conclusion that the dip is a genuine phenomenon of ToM development, and not of interference with some other non-ToM factor, which is not necessarily delayed in children with PDD–NOS. Note that children with autism spectrum disorder are also known to have executive function problems (Blijd-Hoogewys et al., 2014; Craig et al., 2016).

### A Three-Step Developmental Model

Visual inspection of the graphs revealed that girls showed two discontinuities (a plateau and a dip) and boys only one (a dip). The dip of the boys coincided with the second (more shallow) dip of the girls. The dynamic growth model showed that the observable properties of the growth trajectories depend on the values of the parameters governing the growth rate and the supportive and competitive relations between the variables in the model. A typical prediction of the model is that growth rates will result in more clearly observable plateaus and less clearly observable temporary regressions. This prediction is in line with the observed trajectory of the girls: the fact that they show an earlier growth spurt than the boys suggests that the growth rate of their underlying ToM components is higher than that of the boys. Consistent with this presumable higher growth rate, the girls show more clearly observable plateaus and more shallow dips. In short, the proposed dynamic growth model might provide a speculative explanation of the non-linear phenomena observed in the data, including the differences and similarities, the plateaus and dips between boys and girls.

### Limitations of the Research, Prospects for Further Study, and Implications for Clinical Practice

One limitation of our research is that it had fewer children in the older age range (from 8 years on), which implies a reduction in reliability at the older ages. Also, the test was probably too easy for the older children since we did not include more advanced ToM tasks that are typically mastered at later ages. Perhaps additional regressions would have been found at the older ages if second-order belief tasks (Perner and Wimmer, 1985) or more complex emotional constructs would have been used. However, not having included such tasks does not change anything to our main message, that there are non-linearities in ToM development, if it is viewed at from a cross-sectional perspective, with children being confronted with an essentially unfamiliar task, as far as their ToM knowledge is concerned.

A second limitation of our research is that the growth curve of ToM is based on cross-sectional data. This is only one particular perspective on ToM development, namely the perspective provided by asking children to transfer their knowledge to a new and unfamiliar ToM context, namely that of a storybook with explicit verbal questions. Various other complementary perspectives can be provided, for instance that of time-serial frequent measurements or observations of individuals. As is now becoming well-established knowledge, models based on group data should not be seen as models of typical individual curves (see the earlier remark on ergodicity in the introduction, Molenaar and Campbell, 2009). However, there is also converging evidence from longitudinal ToM research both in typically developing children (see Figure 3 in Serra et al., 2002) and children with PDD–NOS (Serra et al., 2002; Blijd-Hoogewys et al., 2010), further supporting the robustness of this developmental phenomenon.

Concerning future research, it might be interesting to include a broader age group, also including second-order and third-order belief tasks. In addition, it might be interesting to focus on directly perceived and enacted forms of other person understanding in the form of micro-observations of social interaction in young children and to compare these implicit forms of understanding with the more explicit forms of understanding that a test like the ToM Storybooks is trying to capture. A third possibility is to focus on the nature of the explanatory schemes that children use or enact while answering questions about desires and intentions. After all, many questions focusing on the understanding of desires and intentions evoke a potential conflict between a scheme of persons as rational-agents (acting on the basis of the real states of affairs in the world) and a scheme of persons as psychological agents (acting on the basis of their knowledge and perception of states of affairs in the world). Of course, these schemes must be coordinated into a scheme of the person as a rational psychological agent, but this process of coordination might not be an easy accomplishment for many children.

The findings of the current research may have implications for clinical assessment and intervention. In the sixth year of life (72–78 months), a dip in ToM understanding and reasoning – in the form of answering explicit questions about imaginary situations – seems common. Note that this is also the age period in which ASD is often diagnosed in children (Miodovnik et al., 2015). Test developers and diagnosticians should take into account that children with ASD may at that time ‘appear’ to have less severe problems on a ToM test if compared to their typically developing peers who are undergoing a temporary ToM dip. Children with ASD show this dip much later (Blijd-Hoogewys et al., 2010). This may appear counterintuitive, for children with ASD do have ToM problems (Baron-Cohen, 2000). Research concerning the impact of the ToM dip on clinical assessment is needed. In individual children, the temporary dip found on the group level might be expressed in the form of temporarily increased intra-individual variability in their reactions to questions involving ToM decisions, for example shifts between direct, rapid, and primarily implicit understanding on the one hand, and reflective, thoughtful and primarily explicit understanding on the other hand, or shifts between rational-agent and psychological-agent perspectives. In principle, clinical interventions might explicitly reckon with the non-linearities in the processes of ToM development, and focus on individual indicators of such non-linearities in the form of rapid learning, resistance to learning, response variability, and so forth, to adapt the intervention to the idiosyncratic nature of the young client’s developmental pathway. Also, ToM training should perhaps focus mainly on acquiring an intuitive and direct way of ToM, only taking into account the cognitive and reflective approach after the dip-age has been reached (Gallagher, 2004; Gallagher and Varga, 2015). How exactly this should be done is of course a matter of further clinical research.

## Conclusion

In sum, this article has explored the existence of non-linearities, in particular temporary regressions, in ToM development. Because little is known about the dynamics in ToM development, a cross-sectional design was applied in combination with non-linear fitting methods. Data from the ToM Storybooks, a comprehensive measurement of ToM, showed that a two or three-step developmental model can be distilled. One non-linearity occurs at the age of 4 years and 8 months (a plateau), and one between the ages of six to six and a half (a dip). These non-linear phenomena could not be explained as accidental sampling effects and were supported by additional indicators of non-linearity, namely changes in skewness, in growth rate, and in variability. The non-linearities, for instance in the form of temporary regressions or dips, were observable not only in the ToM total score, but also in the ToM sub-scores and in both boys and girls. Boys and girls differed somewhat in the form and timing of the non-linear properties. Finally, the dynamic growth models presented in this article might serve as a starting point for the formulation of a theory of ToM in a broader developmental context, focusing on the individual-in-interaction as the locus of the developmental process.

## Author Contributions

Both authors were involved from beginning to end. EB-H is the first author. She gathered the data, did data-analyses and interpretation together with PvG and wrote the article. PvG was responsible for the research design, did data-analyses and interpretation together with EB-H and made revisions to the manuscript. Both authors are in agreement with the content of the manuscript and agree to the byline order and to submission of the manuscript in this form. They agree to be accountable for all aspects of the work in ensuring that questions related to the accuracy or integrity of any part of the work are appropriately investigated and resolved.

## Conflict of Interest Statement

The authors declare that the research was conducted in the absence of any commercial or financial relationships that could be construed as a potential conflict of interest.
