# Supplementary material for: Non-linearities in Theory-of-Mind Development
Source: Front Psychol. 2017 Jan 4;7:1970. doi: 10.3389/fpsyg.2016.01970 (PMC5209372; doi:10.3389/fpsyg.2016.01970)
Supplement: Supplementary file 1 [file Image_1.pdf]

Nonlinearities in Theory-of-Mind development

A simulation sample consisting of 600 individual two-step growth curves with randomized initial level, growth rate and final state level was simulated over 100 time steps (meaning that the age range between 3 and 12 was divided into 100 time points). These simulated curves are of course very different from the empirical data in the sense that they are complete growth curves without any measurement error. However, by taking a random sample at each point in time from all the simulated cases, we simulated a purely cross-sectional measurement, formally similar to the cross-sectional data collected in our ToM study.

Figure 1 shows the pattern of changes in the skewness, first derivatives and standard deviation based on a simulation of a two-step growth process. It shows a series of two peaks in the skewness, the first derivative and the standard deviation. The skewness and first derivative peaks largely coincide (covariance of the series is on average 0.7). The skewness peaks precede the growth rate peaks (first derivative) and the latter precede the variability peaks (standard deviation).

Figure 1. Loess curves of the three qualitative indicators of developmental transition based on simulated data. The skewness peaks precede the growth rate peaks and the latter precede the variability peaks.

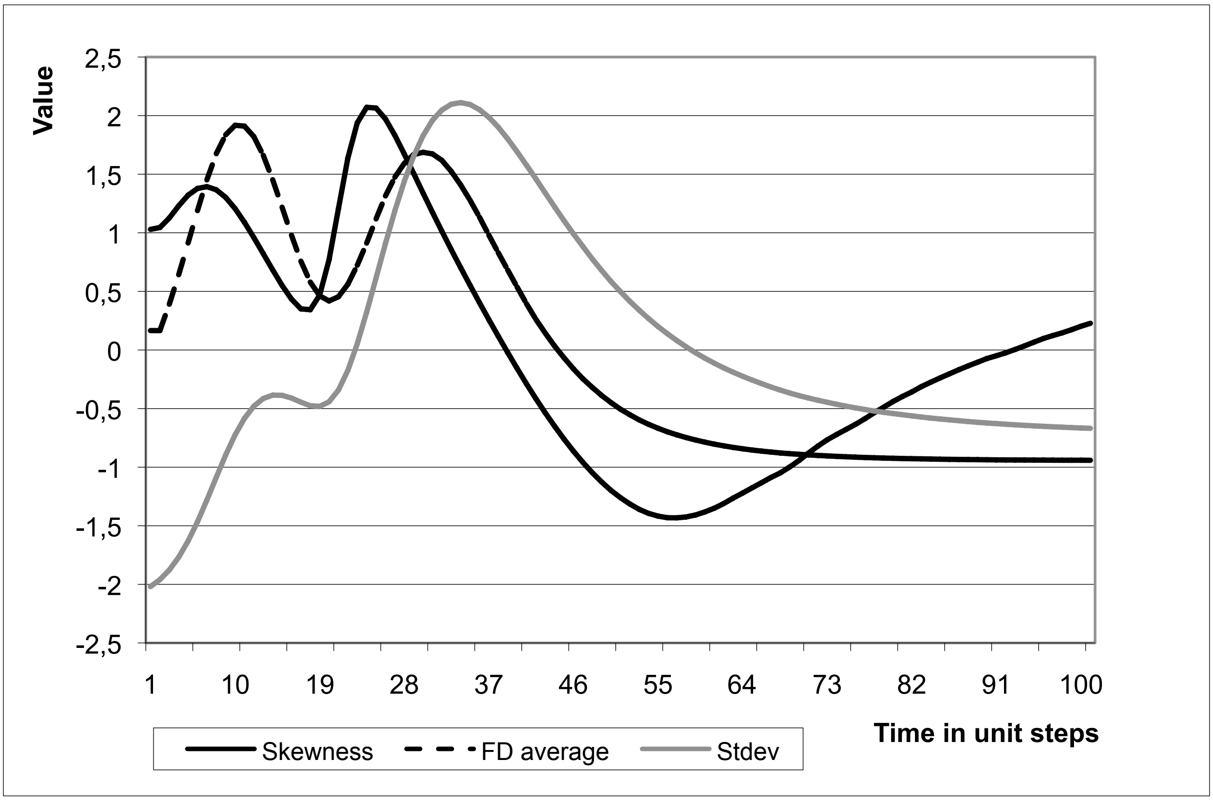

Legend: FD = first derivative (or growth rate), SD = standard deviation (or variability).

In contrast with the simulation data, the empirical data provide only one case for each time point (i.e. each age of a participant). This implies that skewness must be calculated over a moving time window, comprising a sufficient number of cases (we have chosen 21 successive data points, this is averagely a period of 6.34 months). Successive data points are likely to show the progress (or eventually regression) corresponding with their time window (e.g. the period from 34 to 40 months that spans the first time window). Skewness must differ

from the mere change in the variable (if it does not, it adds nothing to our analysis), and thus be defined independent of the change. A good way of doing so is to calculate skewness over the residual values, i.e. the sum-scores minus the corresponding value of the smoothed, nonlinear growth curve. Since the nonlinear smoothing procedure adapts itself optimally to the local averages, the residuals should be expected to be symmetrically divided around zero, except if the skewness varies as a function of developmental time, which is what we expected to find.

After calculating the skewness of the residuals, the skewness data were smoothed by means of a Loess with a 30% window size. First derivatives (i.e. local growth rates) of the developmental curve were then calculated and also smoothed. Lastly, the variability (moving standard deviation) was calculated. The resulting curves are represented in Figure 2 (we used standardized values, so the three criteria can be presented using one scale) (this is Figure 4 in the article).

Figure 2. Loess curves of the three qualitative indicators of developmental transition based on empirical data. A mixture between a two-step and a three-step growth process is obvious. There are two peaks, both in the skewness and in the first derivative curve.

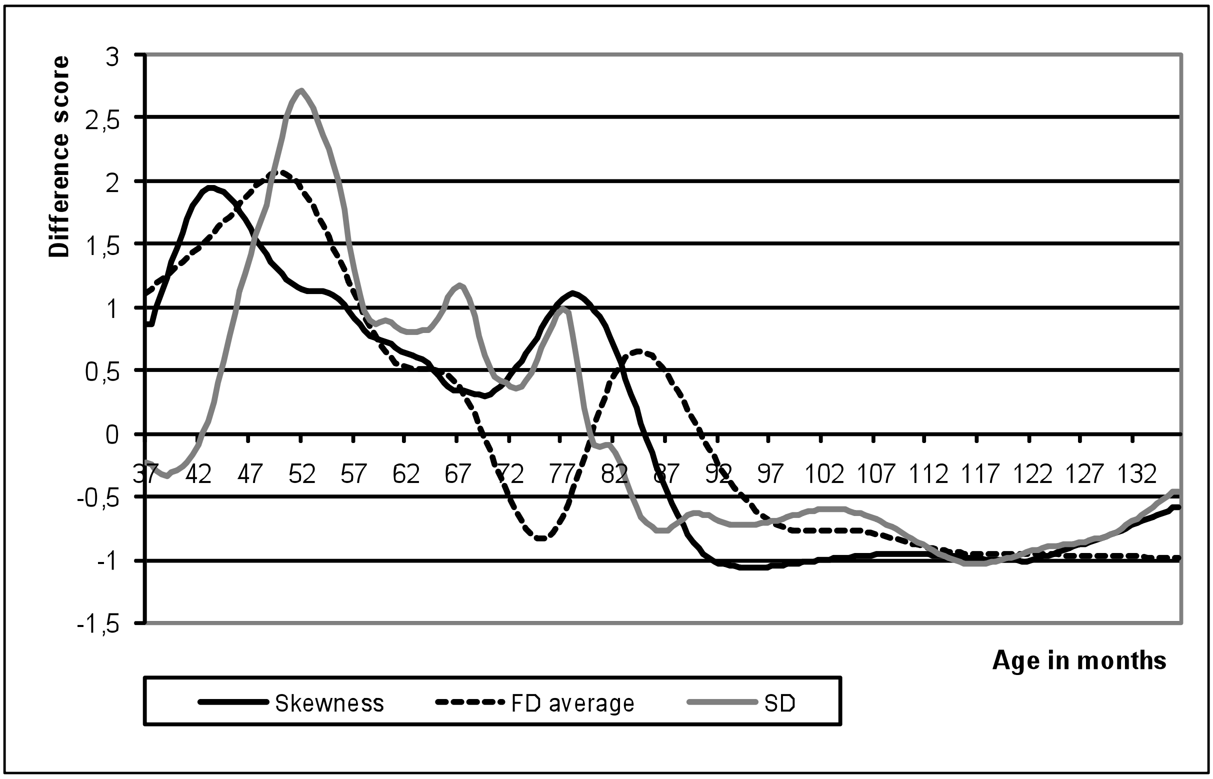

Legend: FD = first serivative (or growth rate), SD =standard deviation (or variability).
